# Complex regional pain syndrome of the knee – a case report

**DOI:** 10.1186/2052-1847-5-12

**Published:** 2013-05-31

**Authors:** Munmun Pandita, Umer Arfath

**Affiliations:** 1 Department of physiotherapy, Sai PG Institute, Dehradun, India; 2 Sardar Bhagwan Singh PG Institute of Biomedical Sciences & Research, Dehradun, India

**Keywords:** Knee pain, Complex regional pain syndrome, Plica

## Abstract

**Background:**

Persistent unexplained pain around the knee can be a perplexing problem. Reports of complex regional pain syndrome involving primarily knee have been published, yet complex regional pain syndrome of the knee is infrequently included in differential diagnosis of pain out of proportion.

**Case presentation:**

A 54 year old female presented to the physiotherapy outpatient department with complains of severe anterior knee pain and stiffness, persisting for more than 2 months post arthroscopic medial plical excision. The patient met the criteria for establishing a probable diagnosis of complex regional pain syndrome (CRPS) knee. Pressure algometre, goniometric measurements and knee outcome survey activities of daily living scale were used to document any changes. This patient was managed for a period of four sessions using graded desensitization therapy, TENS and mobilisation with feedback. Patient showed marked improvement in range of movement (ROM), hypersensitivity, pain and function.

**Conclusion:**

Meticulous examination, early diagnosis and prompt treatment resulted in a quick improvement in the patient’s condition.

## Background

Complex regional pain syndrome (CRPS) is the name now given to group of conditions previously described as reflex sympathetic dystrophy (RSD), causalgia, algodystrophy, sudeck’s atrophy and a variety of other diagnosis [1]. These conditions share a number of clinical features including pain associated allodynia, hyperalgesia, autonomic changes, trophic changes, oedema, and functional loss [2]. Mitchell retrospectively applied the term causalgia to describe a syndrome of burning pain, hyperesthesia, glossy skin and colour changes in limbs of soldiers sustaining major nerve injuries [3]. It was latter recognized that a very similar picture could be produced by a variety of other illnesses & injuries which did not include major nerve injury [2]. According to previous ‘International Association for Study of Pain’ (IASP) definitions of causalgia & RSD, causalgia referred to syndrome associated with nerve injury, while RSD included patients whose pain and associated features followed a variety of insults, most commonly relatively minor & normally fully recoverable injuries. Currently the disease pattern is referred to as complex regional pain syndrome (CRPS). Two types are recognised: CRPS type I without nerve injury and CRPS type II associated with major nerve injury. IASP defines CRPS as a syndrome characterized by a continuing (spontaneous and/or evoked) regional pain that is seemingly disproportionate in time or degree to the usual course of pain after trauma or other lesion. The pain is regional (not in a specific nerve territory or dermatome) and usually has a distal predominance of abnormal sensory, motor, sudomotor, vasomotor edema, and/or trophic findings [4].

The aetiology of CRPS is not fully understood but involves an exaggeration of physiological responses and is now believed to occur on multiple levels within the central nervous system [5]. Prompt diagnosis and early treatment is most effective in altering the course of the disease [6], however making a definite diagnosis is difficult as no imaging or diagnostic modalities are specific for CRPS [7].

Most clinical series of CRPS have either intermingled patients with affected upper & lower extremity or have discussed characteristic management of upper limb only. Though reports involving primarily the knee have been published [5,6,8], a general awareness of the syndrome involving the knee still needs to be increased, and considered in differential diagnosis, so that cases which arise following trauma or otherwise are readily recognized.

## Case presentation

A 54 year female reported to the physiotherapy department with complaints of persistent pain at left knee, with more than two month history of stiffness and functional disability. Area of pain described by the patient was anterior and medial aspect of the knee, with characteristic of pain as burning and induced by any mechanical stimulation, including sensory stimulation from clothing.

The patient had a history of sudden onset of anterior knee pain & locking of left knee while getting up a squatting position, two & half months ago. This was followed by extreme limitation of movement and pain during activity. The patient had undergone arthroscopy two days after the inciting event. Medial plical resection was done through arthroscopy. Further reports had revealed synovial hypertrophy in supra-patellar pouch along with degeneration of medial and lateral patellar facets. One month post arthroscopy, patient had history of painful effusion of the knee. Aspiration had been carried out with 15cc of synovial fluid aspirated.

On observation the patient presented with limp while walking, flexed attitude of the knee along with trophic changes of dry and scaly skin. Skin around the affected area was warm but dry, with edema (non pitting nature) around the anterior aspect of the knee. There was allodynic & hyperalgesic pain response to any palpation on anterior and medial aspect of the knee. Patient revealed global patellar mobility loss with restriction of tibiofemoral joint on active and passive movement examination. Muscle power was reduced to grade 3+, on manual muscle testing (MMT), in the available range. Functional ability of the patient was restricted to a larger extent, such that patient had difficulty in ambulation, managing stairs and most of house hold activities were compromised. Patient’s daily activities were restricted to indoors only, as patient demonstrated fear avoidance behaviour.

**Figure 1:**
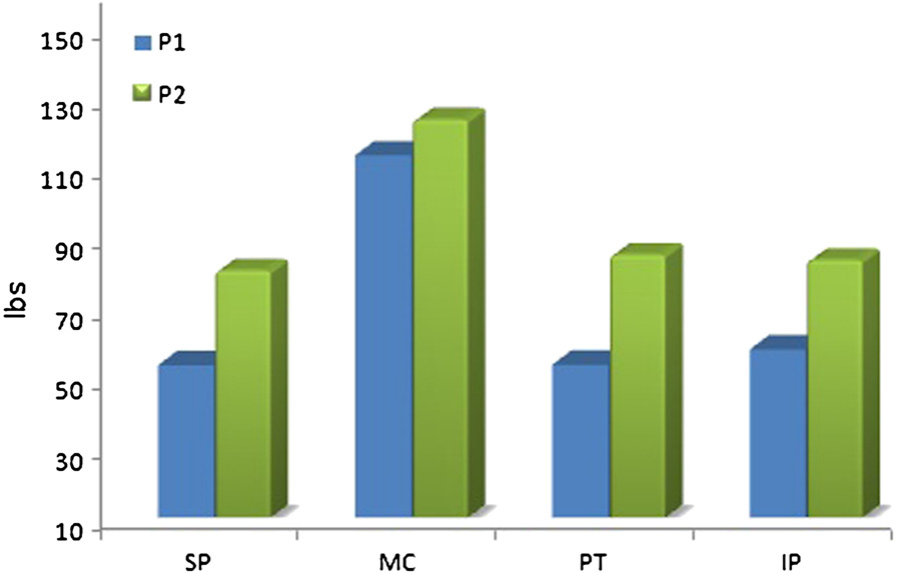
Pressure alogometric measurements pretreatment.

Pressure algometric measurements were carried out for quantification of pain response. Four areas were selected for measurement of algometric readings – supra patellar (SP), medial femoral condyle (MC), centre of patella (PT) & infrapatellar – just superior to tibial tuberosity (IP). The areas were chosen based on area of complaint. Response from three spots from each area was recorded and an average considered. Pain response was recorded as P1 (pressure at onset of pain) & P2 (pressure at maximum pain). Maximum pain response was recorded over supra patellar area followed by infrapatellar, patella and finally by medial femoral condyle (Figure 1). Goniometric measurements of knee recorded an available active range of 20°; from 10° flexion to 30° flexion. Knee outcome survey activities of daily living scale was used for assessing functional limitations of the patient. The scale considers various limitations encountered by the patient in last 1 or 2 days, while performing usual daily activities. It consists of set of 10 questions for with patient is asked to mark the appropriate response. The scale is a reliable, valid and responsive instrument for the assessment of functional limitations that result from wide variety of pathological disorders & impairments of knee [9]. The patient was unable to kneel, squat & sit with knees bent. Severe restriction was recorded while descending from stairs. Ability to rise from chair required use of hands. Walking, associated with limp, and standing ability was less than 10 min.

The patient met the criteria for establishing a probable diagnosis of CRPS knee (type I), after ruling out any post arthroscopic infections, vascular disorders, stress fracture, referred pain, any peripheral neuropathy and any metabolic or inflammatory disorders. In our study the diagnosis was made on clinical grounds using accepted diagnostic criteria [10]. A working hypothesis of CRPS (type I) was established given the reason that patient demonstrated disproportionate pain, hyperalgesia, edema, temperature asymmetry, skin changes, movement loss & absence of major nerve injury.

The patient was managed for a period of four sessions, once per day for 45 min, using graded desensitization therapy, TENS & graded gentle mobilization besides the home program that was taught to the patient.

Transcutaneous electrical stimulation (TENS) was the first modality of choice. TENS was applied using a single channel with electrodes placed at the periphery of the area of complaint i.e. medial condyle, suprapatellar area, lateral border of patella & infrapatellar. Burst TENS was employed using a portable TENS device with a pulse width 100μs, pulse rate of 70 Hz and intensity comfortable for the patient for duration of 20 minutes/day.

Graded desensitization for hyperalgesia was started on the first day of treatment. It included sensory stimulation using various textures. The desensitization was started around the periphery of the lesion with smooth surface first, slowly progressing to a coarser surface and towards the centre of the area of complaint. The desensitization therapy lasted for around 20 minutes each session. Patient was taught and instructed to use desensitization as a home program at least 2 – 3 times a day for 15 minutes duration each. The patient responded well to the treatment and showed good response in terms of tolerance of sensory stimulation.

Gentle mobilization of patella in all directions using Maitland’s grades of oscillatory mobilisation was used [11]. Grade I on 1st day was employed, which progressed to higher grades (grade II & grade III on 2nd & 4th day). Gentle mobilization of patella was also taught to patient, with amplitude as patient tolerated, to be used as home program at least twice a day. Along with patellar mobilization active mobilization of knee joint was performed on every session with 3 – 5 sets and 20 repetitions/set. The patient was instructed to concentrate on gaining maximum ROM with full knee extension. Enough rest time given in between the treatment repetitions to avoid any unnecessary fatigue.

A mirror visual feedback, using the unaffected extremity, was employed for gaining maximum out of active knee mobilization. The patient was instructed to move the affected extremity in relation to unaffected, mirroring its motion both during flexion and extension. The effect of this feedback is based on the finding that visual input from moving, unaffected limb re-establishes pain free relationship between sensory feedback & motor execution of upper limb [12]. Classically this form of treatment is employed using a mirror; we preferred using patient affected extremity itself, as we aimed at gaining maximum ROM.

**Figure 2:**
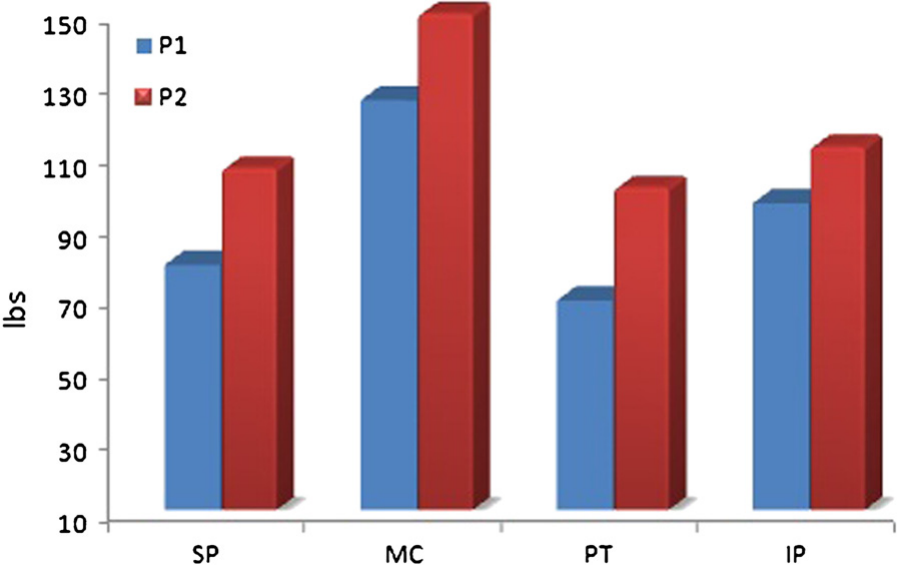
Pressure alogometric measurements after 4 days of treatment.

**Figure 3:**
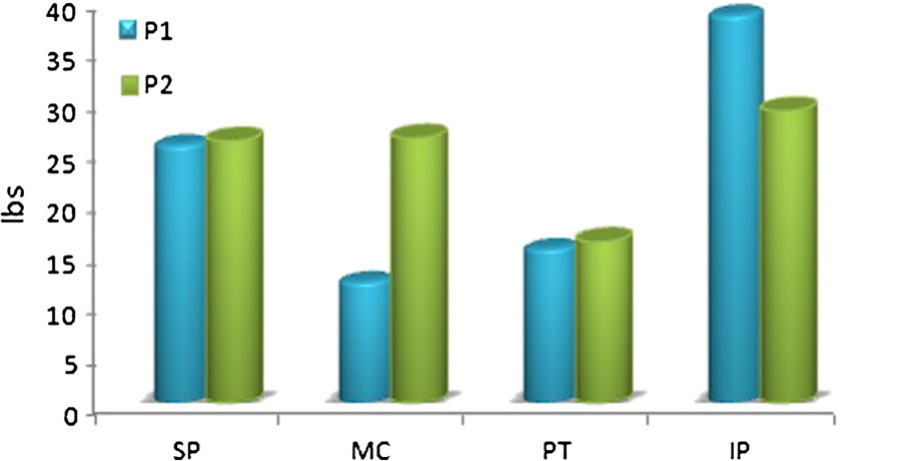
**Change in pain pressure threshold between 1st &4th day.**

Thermotherapy was attempted initially, but patient could not tolerate any form of superficial heating modality well. Patient showed a marked improvement in range of movement (ROM), hypersensitivity, pain and function. The ROM improved from total of 30° pretreatment to 80° after 4 days of treatment. Post 4 days treatment goniometric measurement revealed an active range of 20° flexion to 100° flexion in open chain. Algometric pain responses improved considerably in increased threshold for both P1 & P2. The improvement was seen in all 4 areas with maximum improvement seen in infrapatellar area followed by suprapatellar and medial femoral condylar area. The patellar area pain response improved to the least (Figure 2 and 3).

After 4th day the patient was referred to local physiotherapy OPD for further treatment and was instructed to continue the home program already explained.

## Discussion

CRPS is not a disease, rather a pathological exaggeration of a physiological response, possibly due to misinterpretation and malprocessing of sensory information [13]. Pain severity (often burning) out of proportion to the preceding injury and its persistence is the clue. Other typical features include hyperaesthesia, vasomotor changes, hyperhydrosis, and trophic changes. It occurs at all ages, in women more than men, and the incidence increases until late middle age. Hand and foot involvement is well recognised and this may spread proximally [14]. Conditions affecting the knee frequently do not present with the classic combination of signs and symptoms seen in the upper extremity [15]. The incidence of CRPS after knee surgery is not well appreciated. Evidently there is a wide discrepancy for interpretation of the symptoms and signs necessary to make the diagnosis of CRPS. However a recent study reported that 21% of primary knee arthroplasty patients fulfilled the criteria for the diagnosis one month after the operation, 13% after 3 months, and 12.7% after 6 months [16]. Pathogenesis is poorly understood. It is believed that sensitised wide range multireceptive neurones in the spinal internuncial neuronal pool are at the centre of an abnormal reflex, resulting in excessive sympathetic outflow. For unknown reasons sensitisation occurs after initial nociceptive afferent stimulation, which subsequently results in abnormal pain perception and increased sympathetic afferent activity [17]. Early observations led to widespread acceptance that the sympathetic nervous system is crucially involved in pathogenesis and maintenance of these syndromes [2]. In several large retrospective series trauma, surgical procedures, neurologic disorders and medical conditions were reported to possibly trigger the syndrome. Trauma is the most common precipitant, accounting for 80% of cases, neurological disease accounting for 20% [18]. According to Plewes LW sympathetic dystrophy will occur to some extent in one of every 2000 accidents involving an extremity [19]. Despite several excellent reviews on CRPS in recent years, the disorder is seldom included in differential diagnosis of the painful knee [20].

The extent of symptoms and signs required to make a definitive diagnosis of CRPS is unclear. Patients classically exhibit greater than expected pain with stiffness and slow progress in the absence of component malposition, infection, or other postoperative complications. Vasomotor and sudomotor changes may be difficult to interpret following surgical procedures, especially in the early postoperative period, or trauma if being an inciting event. As with many medical syndromes, patients rarely present all of the classic diagnostic features and unequivocal diagnosis can be difficult and relies predominantly on clinical signs and symptoms [21]. No laboratory test is specific for the diagnosis of CRPS [13]. So, it becomes even more important for a therapist to appreciate the prevelance of problem

A wide variety of therapies have been recommended for treatment of CRPS. Treatment usually requires a multimodal approach, including medications physical and cognitive therapy [13]. The most effective preventative measure is efficient control of pain and as early mobilization as possible. It is generally agreed that an important factor in the effective treatment of CRPS is early recognition and treatment since patients with long-standing duration of disease are less likely to respond well [22]. Several treatments have isolated reports of success. Among these are physical therapy [23], corticosteroids [24] and transcutaneous nerve stimulation [25] have been shown to be very successful. Our management protocol aimed at decreasing the sensitization, increasing range of motion and functional restoration. This approach proved extremely beneficial to the case in discussion with quicker restoration of function.

## Conclusion

CRPS should be considered early in cases of knee injury in any patient who demonstrates disproportionate pain with slower than expected recovery. Early diagnosis and treatment appears to mitigate against poor results and unsuccessful outcomes. Some patients with early CRPS may, however, have spontaneous resolution of their disease [14]. Attempts to prevent the syndrome with early limb mobilization after trauma seem reasonable.

### Consent

Written informed consent was obtained from the patient for the publication of this case report, without any images.

## Competing interests

The authors declared that they do not have any competing interests.

## Authors’ contributions

All authors contributed to the paper and have approved the manuscript.

## Pre-publication history

The pre-publication history for this paper can be accessed here:

http://www.biomedcentral.com/2052-1847/5/12/prepub
